# Incident *Mycobacterium tuberculosis* infection in household contacts of infectious tuberculosis patients in Brazil

**DOI:** 10.1186/s12879-017-2675-3

**Published:** 2017-08-18

**Authors:** Edward C. Jones-López, Carlos Acuña-Villaorduña, Geisa Fregona, Patricia Marques-Rodrigues, Laura F. White, David Jamil Hadad, Lucilia Pereira Dutra-Molina, Solange Vinhas, Avery I. McIntosh, Mary Gaeddert, Rodrigo Ribeiro-Rodrigues, Padmini Salgame, Moises Palaci, David Alland, Jerrold J. Ellner, Reynaldo Dietze

**Affiliations:** 1 Section of Infectious Diseases, Department of Medicine, Boston Medical Center and Boston University School of Medicine, 650 Albany Street, Room 605, Boston, MA 02118 USA; 2 Núcleo de Doenças Infecciosas, Universidade Federal do Espírito Santo (UFES), Vitória, Brazil; 3 Department of Biostatistics, Boston University School of Public Health, Boston, MA USA; 4 Mycobacteriology Laboratory, Núcleo de Doenças Infecciosas, UFES, Vitória, Brazil; 5 Cellular and Molecular Immunology Laboratory, Núcleo de Doenças Infecciosas, UFES, Vitória, Brazil; 6 Division of Infectious Diseases, Department of Medicine, New Jersey Medical School- Rutgers University, Newark, NJ USA

**Keywords:** Household contact study, *M. tuberculosis* infection, TST conversion, Brazil, Latent tuberculosis infection

## Abstract

**Background:**

In household contact investigations of tuberculosis (TB), a second tuberculin skin test (TST) obtained several weeks after a first negative result consistently identifies individuals that undergo TST conversion. It remains unclear whether this delay in *M. tuberculosis* infection is related to differences in the infectious exposure, TST boosting, partial host resistance, or some other factor.

**Methods:**

We conducted a household contact study Vitória, Brazil. Between 2008 and 2013, we identified culture-positive pulmonary TB patients and evaluated their household contacts with both a TST and interferon gamma release assay (IGRA), and identified TST converters at 8–12 weeks post study enrollment. Contacts were classified as TST-positive (≥10 mm) at baseline, TST converters, or persistently TST-negative. We compared TST converters to TST-positive and to TST-negative contacts separately, using generalized estimating equations.

**Results:**

We enrolled 160 index patients and 838 contacts; 523 (62.4%) were TST+, 62 (7.4%) TST converters, and 253 (30.2%) TST−. TST converters were frequently IGRA− at 8–12 weeks. In adjusted analyses, characteristics distinguishing TST converters from TST+ contacts (no contact with another TB patient and residence ownership) were different than those differentiating them from TST− contacts (stronger cough in index patient and contact BCG scar).

**Conclusions:**

The individual risk and timing of *M. tuberculosis* infection within households is variable and dependent on index patient, contact and environmental factors within the household, and the surrounding community. Our findings suggest a threshold effect in the risk of infection in humans.

**Electronic supplementary material:**

The online version of this article (doi:10.1186/s12879-017-2675-3) contains supplementary material, which is available to authorized users.

## Background

Projections to eliminate tuberculosis (TB) by 2050 require mass preventive therapy, which is only possible to implement if individuals with latent TB infection (LTBI) at risk of developing disease are targeted [1]. The disease cycle offers an opportunity for this to be accomplished, as the risk of progression to TB disease is highest in the first 1–2 years after *Mycobacterium tuberculosis* infection is established [2, 3]. After this period, the organism enters into a quiescent state with a low but lifelong risk of exiting dormancy. Therefore, the simple task of timing infection would constitute an incomplete, yet significant step enabling targeted LTBI treatment.

Predictive biomarkers are most needed for household contacts of patients with pulmonary TB, as they are an important focus for screening and prevention programs [4, 5]. However, whereas the overall risk of infection in household contacts is high, their individual probability is variable and dependent on a complex web of host, bacterial and environmental factors [6–8]. The question of whether the observed variability in infection outcomes reflect a greater propensity to generate infectious aerosols by certain index patients [9], strain-specific differences in bacterial virulence [8], partial or complete host resistance to infection [10], or simply environmental factors [8] remains unanswered.

Epidemiologically and programmatically, it is useful to separate household contacts into one of three groups on the basis of their tuberculin skin test (TST) or interferon gamma release assay (IGRA) results [11]. The first two are contacts that are considered infected or not at the time the initial evaluation is performed. Importantly, contacts that are TST-positive or IGRA-positive at initial ascertainment may have been infected by the household member with TB prior to evaluation (i.e. early TST conversion), or in many settings, infected in the community at some time in the past [12]. Because neither TST nor IGRA distinguishes time of infection, this mix of individuals with recent vs. remote infections –and their accompanying variable risk of progression to disease- are aggregated into a single risk group [2, 3, 11]. A third group of contacts can only be identified when placing a second test, usually weeks after the initial test is negative, to identify individuals that undergo TST or IGRA conversion [11, 13].

Beyond adding to the totality of contacts that may benefit from LTBI treatment, the identification of TST converters is important because they are thought to represent a homogeneous group at high risk of progression to disease [2, 3, 14], and because they are immunologically unique as a result of demonstrating acute *M. tuberculosis* infection [15] –akin to the crucial pathogenic insights provided by HIV seroconverters early in the epidemic [16]. Most studies of TST conversion are surveys in populations [17] or surveillance programs in health care workers [2, 3]. However, in neither design is the exposure explicitly identified or conversion measured shortly after infection is established. The aim of this study was to describe individual characteristics of a cohort of household contacts with TST conversion and compare them to contacts that were TST-positive at baseline, and those that remained persistently TST-negative. We hypothesized that TST converters represent the tail end of infections transmitted by the index TB patient within the household and thus, that they were more likely to share characteristics with contacts that were TST-positive at baseline.

## Methods

### Study population

This study was conducted at the Núcleo de Doenças Infecciosas (NDI) located in Vitória, the capital city of the State of Espírito Santo, Brazil. The NDI has organized a network of 16 TB clinics in the metropolitan region of Vitória [9]. In Espírito Santo, the TB incidence is 38/100,000 inhabitants, and the prevalence of HIV infection in the general population is <1, and 7% in TB patients [18].

### Participants

All consecutive pulmonary TB patients attending NDI clinics were eligible, provided they had: 1) age ≥ 18 years with cough ≥3 weeks; 2) first TB episode with ≥1 sputum sample with acid-fast bacilli (AFB) ≥2+ with subsequent *M. tuberculosis* growth in culture; and 3) ≥3 household contacts. We excluded TB patients that were HIV-infected (or refused testing), had a history of TB treatment, or who were too ill to consent, unable to understand, or to comply with the study protocol. A household contact was defined as an individual of any age fulfilling one of the following culturally-adapted criteria of close contact with the index patient for ≥3 months before enrollment: 1) sleeping under the same roof ≥5 days/week; 2) sharing meals ≥5 days/week; 3) watching TV nights on weekends, and; 4) other significant contact (85% visited the household ≥18 days/month). To minimize differences in exposure time, participants were enrolled within the first 2 weeks after the index patient presented to the clinic.

### Measurements

*Index TB* patients. We collected clinical information and measured cough severity using a self-reported visual analog cough scale (VACS) and the Leicester Cough Questionnaire (LCQ), as reported [8]. We obtained up to three sputum specimens for AFB smear (auramine O fluorescent stain) and culture (Ogawa-Kudoh method) [19]. The radiological extent of disease was graded on a four-category ordinal scale by an experienced radiologist [20]. All patients were offered TB treatment according to Brazilian guidelines [21]. *Household contacts*. We followed Brazilian recommendations for household contact investigations [21]. Study staff visited dwellings to record clinical information, measure individual contact time with the index patient, and to perform an environmental evaluation (crowding and ventilation). BCG vaccination status was assessed by the presence of a scar in the deltoid area. Contacts with TST ≥10 mm and secondary TB suspects were referred for evaluation to the corresponding TB Clinic.

#### TST and IGRA testing and retesting protocol

We evaluated contacts for *M. tuberculosis* infection with both TST and IGRA (Quantiferon Gold-In-Tube, Quiagen, U.S.A.), as described [22]. Briefly, only trained staff performed TST, and we completed TST reading evaluations (kappa >90%) prior to opening study enrollment. Two units of R23 (SSI, Denmark) were placed on the forearm of contacts using the Mantoux method, and the diameter of induration measured in millimeters between 72 and 96 h. Contacts with a TST < 10 mm at baseline were retested after 8–12 weeks to identify TST conversion, using two different criteria: *Criterion 1* (Brazilian Guideline): 1st TST **<** 10 mm, 2nd TST ≥10 mm, and Δ between 1st and 2nd TST ≥10 mm. *Criterion 2*: 1st TST <5 mm, 2nd TST ≥10 mm, and Δ ≥6 mm [13]. IGRA testing was done at 8–12 weeks after the initial TST. For contacts in whom a second TST was needed (initial TST <10 mm), blood for IGRA was obtained before placement of the second TST to minimize boosting [23].

### Statistical methods

Three groups of household contacts were identified for the outcome analysis: 1) TST ≥10 mm at baseline; 2) TST converters (Criterion 1 or Criterion 2), and; 3) TST <10 mm at 8–12 weeks. We summarized index patient and contact characteristics for each of the TST-defined groups. We compared TST-converters to TST-positive at baseline (primary comparison), and separately to TST-negatives (secondary comparison) using bivariate logistic regression models fit using generalized estimating equations (GEE). Variables with *p* < 0.1 in univariate analyses and those deemed clinically important were included in multivariable GEE models. We also summarize TST and IGRA readings on contacts by age groups (0–5, 6–10, 11–20, 21–40, >40 years old). All analyses were performed using SAS version 9.3 (SAS Institute, Cary, NC) and R version 3.3.1 (www.r-project.org).

#### Ethical approvals

The study was approved by the Comitê de Ética em Pesquisa do Centro de Ciências da Saúde - Universidade Federal do Espírito Santo and the Comissão Nacional de Ética em Pesquisa, and the Institutional Review Boards of Boston University Medical Center and Rutgers University –New Jersey Medical School (formerly University of Medicine and Dentistry of New Jersey). We obtained written informed consent and assent in Portuguese in accordance with age-specific ethical guidelines of participating institutions.

## Results

**Fig. 1:**
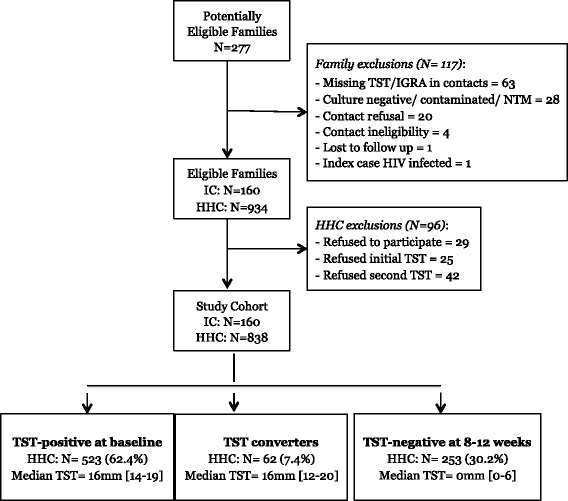
Study profile

Between February 2008 and October 2013, we identified 277 potentially eligible families but excluded 117 of them (Fig. 1). Of the remaining 160 index patients and their 934 household contacts, we excluded 96 contacts because of incomplete TST/IGRA results (*N* = 67) or refusal to participate (*N* = 29). The 67 excluded contacts were more likely to be male (57 vs. 43%, *p* = 0.03) compared to those included but were otherwise similar in terms of age (*p* = 0.74) and BCG scar (*p* = 0.99). Therefore, this analysis included 160 index TB patients and 838 household contacts; 523 (62.4%) contacts were TST-positive (≥10 mm) at baseline, 62 (7.4%) were TST converters, and 253 (30.2%) remained TST-negative (<10 mm) at study conclusion.

### Description of households

Table 1 and Additional file 1: Table S1 show characteristics of household contacts, index TB patients and study dwellings in the three TST-defined groups of contacts. Overall, contacts were young (median 21 years, interquartile range [IQR] 10–39), female (57%), BCG vaccinated (82%) and HIV-uninfected. Contacts were heavily exposed to TB both inside and outside the household, as 20% had contact with another TB patient in the past. Index TB patients were young adults (median 36 years, IQR 23–42), male (66%) with advanced TB disease. Study dwellings were crowded (median 6 contacts), and 14% of families lived in a rented home, an indicator of lower socio-economic status in this setting.

**Table 1:** Characteristics of household contacts, index tuberculosis cases and study dwellings according to the tuberculin skin test (TST) in contacts at study completion (8–12 weeks after enrollment)

Variable	All (*N* = 838)	TST-positive at baseline (*n* = 523)	TST converters (*n* = 62)	OR 95% CI	P +	TST-negative (*n* = 253)	OR 95% CI	P ++
Contact Factors								
Age (per 10 years)	20 [10–39]	22 [12–41]	15 [10–39]	0.98 (0.76–1.01)	0.06	18 [8–34]	1.01 (0.90–1.15)	0.77
Gender					0.15			0.07
Male	365 (44)	231 (44)	21 (34)	Ref.		113 (45)	Ref.	
Female	473 (56)	292 (56)	41 (66)	1.38 (0.89–2.14)		140 (55)	1.63 (0.96–2.78)	
BCG scar					0.18			0.09
No/ uncertain	152 (18)	98 (18)	8 (13)	Ref		46 (19)	Ref	
Yes	686 (82)	425 (81)	54 (87)	1.61 (0.80–3.24)		207 (82)	1.99 (0.89–4.48)	
Other TB contact^a^					0.01			0.92
No	665 (80)	389 (75)	56 (90)	Ref		220 (87)	Ref	
Yes	166 (20)	127 (25)	6 (10)	0.38 (0.18–0.80)		33 (13)	0.94 (0.40–2.25)	
Index factors								
Age (years)	36 [23–42]	34 [23–44]	32 [22–39]	0.99 (0.96–1.01)	0.39	37 [27–42]	0.98 (0.95–1.00)	0.08
Cough duration (weeks)	8 [4–16]	8 [4–16]	8 [4–12]	0.96 (0.92–1.02)	0.17	8 [4–16]	0.97 (0.94–1.00)	0.09
VACS	8 [5–9]	8 [5–9]	9 [8–10]	1.02 (0.89–1.17)	0.74	5 [3–8.5]	1.24 (1.08–1.42)	0.003
LCQ	12 [10–16]	11 [10–14]	12 [10–15]	0.97 (0.87–1.07)	0.51	13 [11–17]	0.87 (0.79–0.97)	0.01
Extent of disease on chest radiograph								
Normal/Minimal	49 (6)	22 (4)	11 (18)	Ref.	0.15	16 (6)	Ref.	0.66
Moderate	406 (49)	235 (46)	31 (50)	0.50 (0.15–1.63)		140 (56)	0.58 (0.14–2.08)	
Advanced	371 (45)	257 (50)	20 (32)	0.32 (0.10–1.06)		94 (38)	0.55 (0.14–2.17)	
Cavitation								
No	211 (26)	102 (20)	22 (35)	Ref		87 (35)	Ref	
Yes	615 (74)	412 (80)	40 (65)	0.51 (0.24–1.11)	0.09	163 (65)	1.10 (0.50, 2.41)	0.81
Environmental Factors								
Own residence					0.06			0.17
No	115(14)	98 (19)	2 (3)	Ref		15 (6)	Ref	
Yes	723 (86)	425 (81)	60 (97)	4.22 (0.96–18.6)		238 (94)	2.44 (0.69-8.61)	
Sleep in same room as index					0.34			0.004
No	302 (36)	170 (33)	16 (26)	Ref		116 (46)	Ref	
Yes	536 (64)	353 (67)	46 (74)	1.42 (0.59–3.4)		137 (54)	1.35 (1.45-7.35)	
Contact time with index (hours/ day)					0.31			0.04
≤ 6 h	255 (31)	142 (27)	16 (26)	Ref		97 (38)	Ref	
> 6 h	578 (69)	376 (73)	46 (74)	1.31 (0.78–2.20)		156 (62)	1.83 (1.02–3.27)	

*Definition of abbreviations*: *AFB* Acid-Fast Bacilli, *BCG* Bacille Calmette-Guérin vaccine, *LCQ* Leicester Cough Questionnaire, *TB* tuberculosis, *VACS* Visual Analog Cough Scale, *SD* Standard deviation

Values are median [Interquartile range] or number (percentage), unless otherwise specified

Missing data: Other TB contact (7), hours per day of contact (5)

+ Univariate analysis using generalized estimating equations comparing TST converters vs. TST-positive at baseline

++ Univariate analysis using generalized estimating equations comparing TST converters vs. TST-negative at 8–12 weeks

^a^ Other TB contact refers to known contact with another person with TB outside household

For age, OR estimated as the ratio of odds per 10 year increase

Contacts that were TST-positive at baseline had a median age of 22 years (IQR 12–41), 56% were female, 81% had a BCG scar, and 96% of their index TB patients had moderately or far-advanced disease on chest x-ray, including 80% with cavitations (Table 1); 25% of contacts in this group had contact with a TB patient outside the household, and 19% lived in a rented home. In comparison, contacts with TST conversion were younger (median 15 years, IQR 10–39), more frequently female (67%) and BCG vaccinated (87%), and their index TB patients had less severe disease (82%) and less cavitations (65%) on chest radiograph; only 10% had contact with another patient, and 3% rented their residence. Overall, contacts that remained TST-negative had a less intense infectious exposure when compared to the other two groups of contacts.

### TST/IGRA results in household contacts

**Fig. 2:**
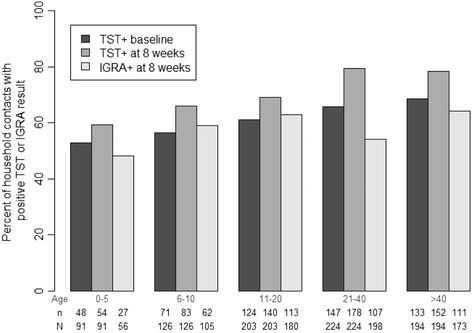
Tuberculin skin test (TST) and interferon gamma release assay (IGRA) results at baseline and 8–12 weeks after enrollment according to contact age in 838 household contacts of pulmonary tuberculosis cases in Vitória, Brazil

Table 2 shows a qualitative and quantitative analysis of TST and IGRA readouts in contacts. Of the 421/523 TST-positive contacts and 58/62 TST converters with available IGRA results, the former were more likely to be IGRA-positive at 8–12 weeks (81 vs. 69%, respectively; *p* = 0.04). TST/IGRA agreement was 80% (Kappa 0.56) in all contacts and 69% (Kappa 0.04) in TST converters (*N* = 58). Quantitatively, the median TST induration size (16 mm vs. 16 mm, *p* = 0.93) and median IGRA readouts (4.4 IU/mL vs. 5.92 IU/mL, *p* = 0.75) were similar in both groups. Figure 2 shows a breakdown of TST and IGRA results at baseline and at 8–12 weeks by contact age groups.

**Table 2:** Qualitative and quantitative analysis of tuberculin skin test (TST) and interferon gamma release assay (IGRA) readouts in household contacts according to their TST status at study completion (8–12 weeks after enrollment)

Variable	All (*N* = 838)	TST-positive ^a^at baseline (*n* = 523)	TST converters (*n* = 62)	OR 95% CI	P ^d^	TST-negative (*n* = 253)	OR 95% CI	P ^e^
At Baseline								
TST1 (mm) ^b^								
Median [IQR]	13 [0–17]	16 [14–19]	0 [0–3]			0 [0–2]		
Mean [SD]	11 {7.9}	16 {3.7}	1 {2.3]	−	−	2 {2.9}	−	−
Range	[0–30]	[10–30]	[0–8]			[0–9]		
≥5 mm	576 (69%)	−	−			−		
≥10 mm	523 (62%)	−	−			−		
≥15 mm	349 (42%)	−	−			−		
At 8–12 weeks								
Max TST (mm) ^c^								
Median [IQR]	14 [8–18]	16 [14–19]	16 [12–20]			0 [0–6]		
Mean [SD]	12 {7.3}	16 {3.7}	16 {5.2}	0.98 (0.92–1.05)	0.58	3 {4.3}	−	−
Range	[0–30]	[10–30]	[0–25]			[0–17]		
≥5 mm	656 (78%)	−	−			−		
≥ 10 mm	607 (72%)	−	−			−		
≥ 15 mm	396 (47%)	−	−			−		
IGRA (IU/mL)								
Median [IQR]	1.03 [0.03-8.6]	4.4 [0.68–10]	4.6 [0.07–10]			0.02 [0–0.11]		
Mean [SD]	3.6 {4.2}	5.1 {4.2}	5.0 {4.6}	1.01 (0.95-1.07)	0.75	0.6 {1.9}	1.42 (1.28-1.58)	<0.001
Range	[0–14]	[0–14]	[0–10]			[0–10]		
Negative	292 (41)	78 (19)	18 (31)	1.92 (1.02–3.7)	0.04	196 (84)	−	−
Positive	420 (59)	343 (81)	40 (69)	Ref		37 (16)		

*Definition of abbreviations*: *TST* Tuberculin skin test, *IGRA* Interferon gamma release assay (Quantiferon Gold-In-Tube), *IQR* Interquartile range, *SD* Standard deviation

Values are median [Interquartile range] or number (percentage), unless otherwise specified

Missing data: IGRA (*n* = 126)

^a^ TST ≥10 mm

^b^ TST1 was placed at study entry

^c^ Max TST indicates maximum TST value for each contact (TST1 or TST2)

^d^ Univariate analysis using generalized estimating equations comparing TST converters vs. TST-positive at baseline

^e^ Univariate analysis using generalized estimating equations comparing TST converters vs. TST-negative at 8–12 weeks

Table 3 shows a qualitative and quantitative analysis of TST/IGRA readouts in contacts with TST conversion, stratified by contact age. Whereas the baseline prevalence of TST-positivity increased with age (Fig. 2), the proportion of contacts with TST conversion was similar across age categories (*p* = 0.12). The same analysis using three other definitions for TST conversion found similar results (Additional file 2: Table S2).

**Table 3:** Qualitative and quantitative analysis of tuberculin skin test (TST) and interferon gamma release assay (IGRA) results in household contacts with TST conversion according to contact age

Variable	Total	Household Contact Age Group (years)				
		0–5	6–10	11–20	21–40	>40
Number of contacts	838	91	126	203	224	194
TST <5 mm at baseline (at risk of conversion)	262	42	52	68	53	47
TST conversion (Criterion 1 OR Criterion 2)						
N converters/ total contacts (%)	62 (7.4)	5 (5.5)	12 (9.5)	17 (8.3)	14 (6.3)	14 (7.2)
TST size in converters (mm)						
Median [IQR]	16 [13–20]	11 [10–20]	18 [15–20]	15 [14–18]	18 [10–19]	15 [12–16]
Mean [SD]	16 {4.2}	14 {5.3}	17 {3.7}	16 {3.3}	16 {5.0}	17 {4.6}
Range	[10–25}	[10–20]	[10–23]	[10–24}	[10–24]	[11–25]
IGRA in TST converters (IU/mL)						
Positive (%)	40/58 (69)	2/3 (67)	8/12 (67)	12/16 (75)	8/13 (62)	10/14 (71)
Median [IQR]	4.7 [0.07–10]	6.4 [0–10]	10.3 [0.07–10]	10 [0.08–10]	2.6 [0.04–9.1]	2.7 [0.2–10]
Mean [SD]	5.1 {4.6}	5.5 {5.1}	5.1 {4.7}	6.2 {4.7}	4.2 [4.8]	4.5 {4.5}
Range	[0–10]	[0–10]	[0–10]	[0–10]	[0–10]	[0–10]

*Definition of abbreviations*: *IGRA* Interferon gamma release assay (Quantiferon Gold-In-Tube), *IQR* Interquartile range, *SD* Standard deviation

*Criterion 1* (Brazilian Guidelines): 1st TST **<** 10 mm, 2nd TST ≥10 mm, and difference between 1st and 2nd TST ≥10 mm

*Criterion 2*: 1st TST <5 mm, 2nd TST ≥10 mm, and difference between 1st and 2nd TST ≥6 mm

Four TST converters had missing values for IGRA: 0–5 years (2), 11–20 years (1), 21–40 years (1)

IGRA value is at time of TST conversion (8–12 weeks)

### Comparison of TST converters vs. TST-positive contacts at baseline

In an unadjusted analysis that compared TST converters vs. TST-positive contacts (Table 1 and Additional file 1: Table S1), the former were less likely to have contact with another TB patient outside of the household (odds ratio [OR] 0.38, 95% confidence interval [CI] 0.18–0.80; *p* = 0.01) and more likely to be IGRA-negative at 8–12 weeks (OR 1.92, 95% CI 1.02–3.7; *p* = 0.04). In an adjusted analysis (Table 4), TST conversion was associated with no contact with a TB patient outside the household (OR 0.36, 95% CI 0.16–0.82; *p* = 0.01) and residence ownership (OR 4.87, 95% CI 1.05–22.5; *p* = 0.04), and marginally associated with having a negative IGRA at 8–12 weeks (OR 1.73, 95% CI 0.98–3.07; *p* = 0.06) and contact female gender (OR 1.58, 95% CI 0.96–2.60; *p* = 0.07).

**Table 4:** Multivariable analysis of factors associated with tuberculin skin test (TST) positivity, conversion or negativity in 838 household contacts in Vitória, Brazil

Variable	TST-positive at baseline (*n* = 523)	TST Converter (*n* = 62)	OR^a^ (95% CI)	P +	TST negative (*n* = 253)	OR^b^ (95% CI)*	P ++
Contact Factors							
Age (years)	22 [12–41]	15 [10–39]	0.99 (0.97–1.00)	0.17	18 [8–34]	1.01 (0.99–1.03)	0.24
Female	292 (56)	41 (66)	1.58 (0.96–2.60)	0.07	140 (55)	1.70 (0.95–3.06)	0.07
BCG scar present	425 (81)	54 (87)	1.58 (0.96–2.60)	0.32	207 (82)	2.96 (1.01–8.67)	0.05
Other TB contact	127 (25)	6 (10)	0.36 (0.16—0.82)	0.01	33 (13)	-	-
IGRA negative	78 (19)	18 (31)	1.73 (0.98–3.07)	0.06	196 (84)	-	-
Index Factors							
Age (years)	34 [23–44]	32 [22–39]	-	-	37 [27–42]	0.98 (0.94–1.01)	0.15
Cough duration (weeks)	8 [4–16]	8 [4–12]	0.96 (0.91–1.02)	0.16	8 [4–16]	0.97 (0.93–1.00)	0.08
VACS	8 [5–9]	9 [8–10]	-	-	5 [3–8.5]	1.20 (1.03–1.40)	0.02
Extent of disease on CxR^1^	257 (50)	20 (32)	0.87 (0.40–1.94)	0.74	945 (38)	-	-
Presence of cavities	412 (80)	40 (65)	0.68 (0.22–2.05)	0.49	163 (65)	0.96 (0.40–2.29)	0.93
Environmental Factors							
Own residence	425 (81)	60 (97)	4.87 (1.05–22.5)	0.04	238 (94)	-	-
Sleep in the same room	353 (67)	46 (74)	-	-	137 (54)	2.22 (0.91–5.36)	0.08
> 6 h / day of contact	376 (73)	46 (74)	-	-	156 (62)	1.37 (0.64–2.95)	0.42

*Definition of abbreviations*: *BCG* Bacille Calmette-Guérin vaccine, *CI* Confidence interval, *CxR* Chest x-ray, *IGRA* Interferon gamma release assay (Quantiferon Gold-In-Tube), *OR* Odds ratio, *TB* Tuberculosis, *VACS* Visual Analog Cough Scale

Values are median [Interquartile range] or number (percentage), unless otherwise specified

^a^Odds ratio and 95% CI estimated for TST converters when compared to TST-positive at baseline. For numeric variables, odds ratio represent odds of being a TST converter per 1 unit of increase of the variable

^b^Odds ratio and 95% CI estimated for TST converters when compared to TST-negative at 8–12 weekss. For numeric variables, odds ratio represent odds of being a TST converter per 1 unit of increase of the variable

+ Estimated using multivariate GEE to compare risks of being TST converters vs. TST-positive at baseline. Model is adjusted for other variables listed in the corresponding table column

++ Estimated using conditional multivariate GEE to compare TST converters vs. TST-negative at 8–12 weeks. Model is adjusted for other variables listed in the corresponding table column

OR estimated comparing far advanced disease versus normal/minimal disease on chest radiograph

### Comparison of TST converters vs. persistently TST-negative contacts

In an unadjusted analysis that compared TST converters vs. TST-negative contacts (Table 1 and Additional file 1: Table S1), TST converters were more likely to have an index TB patient with more severe cough as measured by higher VACS scores (OR 1.24, 95% CI 1.07–1.42; *p* = 0.003) or lower LCQ scores (OR 0.87, 95% CI 0.79–0.97; *p* = 0.01), to sleep in the same room as the index patient (OR 1.35, 95% CI 1.45–7.35; *p* = 0.004), and to have ≥6 h/day of contact with the index patient (OR 1.83, 95% CI 1.02–3.27; *p* = 0.04). In an adjusted analysis (Table 4), TST conversion in contacts was associated with a higher VACS score in the index patient (OR 1.20, 95% CI 1.03–1.40; *p* = 0.02) and BCG vaccination in the contact (OR 2.92, 95% CI 0.92–9.25; *p* = 0.05), and marginally associated with contact female gender (OR 1.70, 95% CI 0.95–3.06; *p* = 0.07), and sleeping in the same room (OR 2.22, 95% CI 0.91–5.36; *p* = 0.08) and a shorter duration of cough in the index patient (OR 0.97, 95% CI 0.93–1.0; *p* = "0"s are written incorrectly (o.o8) - please correct to 0.08).

## Discussion

In this household contact study in a mostly urban, intermediate TB prevalence setting we found that 7% of contacts underwent TST conversion during the 8–12 week study observation period. Our results show that the individual risk and timing of *M. tuberculosis* infection is variable and modulated by a combination of index patient, contact and environmental factors within the household, and the surrounding community. Although we found differences between households containing infected and non-infected contacts, our findings are consistent with the hypothesis that family units with TST-converters had a similar infectious exposure than contacts that are TST-positive at baseline, when compared to those that remain TST-negative. Our results suggest that certain individual contact factors such as gender and BCG vaccination may impact the timing and nature of *M. tuberculosis* infection within a household environment. We also found that Quantiferon-Gold-In-Tube did not perform well in household contacts with recent *M. tuberculosis* infection as measured by TST conversion, a population known to be at increased risk of progression to TB disease.

In many settings, the risk of acquiring *M. tuberculosis* infection is cumulative throughout life [24, 25], and particularly high in household contacts of pulmonary TB patients as a result of two, or more, infectious exposures by virtue of cohabitating with a person with TB, and living in a community with ubiquitous transmission [12]. However, differentiation between community- vs. household-acquired infection is challenging [4, 12]. Using a mathematical model, Brooks-Pollock et al. inferred that in Peru up to 70% of TST-positive household contacts were due to community transmission [26]. In Uganda, Whalen et al. found that 71% of contacts were infected compared to only 24% of community controls, suggesting predominantly household transmission [12]. Expectably, in the present study contacts that were TST-positive at baseline more frequently had contact with a person with TB outside the household. Ideally, in order to properly control for community based transmission the evaluation of household contacts should begin before the index patient becomes infectious. To our knowledge, such a study has not yet been conducted.

A related issue is our limited understanding of the effect these two components of risk (community vs. household transmission) have on the timing of infection in contacts. In low TB prevalence communities with well-performing TB control programs the likelihood of infection is low in both the community and within households, as a result of early TB patient detection. In such settings, most infections in household contact are likely related to the index patient exposure, and consequently, a significant proportion of contacts would be expected to have TST conversion when evaluated. Conversely, in high prevalence settings, the expected proportion of contacts with TST conversion would be lower as a result of the opposite epidemiologic scenario. Interestingly, the proportion of contacts with TST conversion in the present study is consistent with the 4–10% reported in most other studies in both low and high TB burden settings [15, 27–29]. The narrow variation in TST conversion across vastly different epidemiologic settings is striking in itself and unlikely to be explained by chance alone. We posit this observation suggests a threshold effect in the risk of *M. tuberculosis* in humans where some individuals become infected early when the infectious force is minimal, whereas others only become infected after a more intense or sustained exposure. In a follow-up study that will include immunological studies with stored blood samples from contacts, we will examine if the observed variability in timing or susceptibility to infection is associated with differences in progression to disease during follow-up.

Recently, there has been great interest in studying household contacts that remain uninfected despite close contact with a TB patient, as they are thought to represent a phenotype of innate or acquired resistance to *M. tuberculosis* infection [3, 10]. In meta-analyses of household contact studies ~50% of contacts are found to be TST-negative [4, 5], but this may be due to multiple factors such as exposure misclassification [29], missing TST converters, and limited sensitivity of TST and IGRA [3]. Our findings are in agreement with previous studies that found an association between TST conversion and socioeconomic status, [30] and lower rates of infection in females [27] –possibly as a result of family behavior patterns. We also found a weak association between BCG scar and TST conversion when compared to TST-negative contacts, suggesting BCG may have boosted TST conversion in the former. Given the implications for vaccine design and growing evidence that BCG may prevent *M. tuberculosis* infection [31], future mechanistic studies will be needed to elucidate and expand on these findings.

Our study has limitations. Selection bias may have occurred as the higher proportion of males in the excluded population may have overestimated the effect of female gender in TST conversion. The absence of community controls did not permit estimating the secondary attack rate of TB infection within households [12]. We did not obtain cough generated *M. tuberculosis* aerosols in index patients, which may have led to exposure misclassification [29]. We used the visualization of a scar as a surrogate for BCG vaccination although it is estimated that up to 10% of BCG vaccinated infants may not develop a scar [13]. Finally, we used two definitions for TST conversion to minimize the possibility of TST boosting, which is rarely associated with a TST diameter increase >6 mm.

## Conclusion

Our results suggest TST converters are likely to represent late infections resulting from the index patient exposure but that certain individual contact factors may modulate the timing and nature of infection. Taken together, our findings and those of others suggest the existence of an immunological threshold in certain contacts that become infected only after a sustained or unusually large infectious inoculum resulting from both community and household exposures. If proven to be correct in future studies with immunological and TB disease endpoints, such a finding would have important implications for TB immunopathogenesis and vaccine development.

## Additional files

Additional file 1: Table S1. Additional characteristics of household contacts, index tuberculosis cases and study dwellings according to the tuberculin skin test (TST) outcome in contacts at study completion. (DOCX 20 kb)

Additional file 2: Table S2. Qualitative and quantitative analysis of tuberculin skin test (TST) and interferon gamma release assay (IGRA) results in household contacts with TST conversion according to contact age and additional TST conversion criteria. (DOCX 17 kb)

## Abbreviations

AFB: Acid fast bacilli
BCG: Bacillus calmette-guerin
GEE: Generalized estimating equations
HIV: Human immunodeficiency virus
IGRA: Interferon gamma release assay
LCQ: Leicester cough questionnaire
LTBI: Latent TB infection
NDI: Nucleo de doencas infecciosas
TB: Tuberculosis
TST: Tuberculin skin test
VACS: Visual analog cough scale
