# Supplementary material for: Incident Mycobacterium tuberculosis infection in household contacts of infectious tuberculosis patients in Brazil
Source: BMC Infect Dis. 2017 Aug 18;17:576. doi: 10.1186/s12879-017-2675-3 (PMC5563014; doi:10.1186/s12879-017-2675-3)
Supplement: Supplementary file 1 — Additional characteristics of household contacts, index tuberculosis cases and study dwellings according to the tuberculin skin test (TST) outcome in contacts at study completion. (DOCX 20 kb) [file 12879_2017_2675_MOESM1_ESM.docx]

| **Variable** | **All**  **(N=838)** | **TST-positive at baseline (n= 523)** | **TST converters**  **(n= 62)** | **OR 95% CI** | **P +** | **TST-negative**  **(n= 253)** | **OR 95% CI** | **P ++** |
| --- | --- | --- | --- | --- | --- | --- | --- | --- |
| Contact Factors |  |  |  |  |  |  |  |  |
| BMI (kg/m^2^) | 21.2 [17.2-25.7] | 21.5 [17.6-25.6] | 19.4 [17.3-25.0] | 0.99 (0.94-1.02) | 0.38 | 20.7 [16.6-25.7] | 0.99 (0.95- 1.04) | 0.93 |
| Any smoking  No  Yes | 637 (77)  194 (23) | 375 (73)  142 (27) | 49 (80)  12 (20) | Ref  0.53 (0.26-1.05) | 0.07 | 213 (84)  40 (16) | Ref  1.12 (0.60- 2.11) | 0.71 |
| Index factors |  |  |  |  |  |  |  |  |
| Gender  Male  Female | 557 (67)  281 (33) | 333 (64)  190 (36) | 43 (69)  19 (31) | Ref  0.90 (0.4-2.01) | 0.80 | 181 (72)  72 (28) | Ref  1.14 (0.50-2.59) | 0.75 |
| BMI (kg/m^2^) | 19.8 [18-22] | 19.6 [18-21.9] | 21.7 [18.7-23.4] | 1.04 (0.97-1.12) | 0.23 | 19.8 [17.7-22.1] | 1.03 (0.95, 1.11) | 0.44 |
| Sputum AFB  2+  3+ | 168 (20)  670 (80) | 99 (19)  424 (81) | 12 (19)  50 (81) | Ref  1.40 (0.5-3.87) | 0.52 | 57 (23)  196 (77) | Ref  1.78 (0.61-5.19) | 0.29 |
| Environmental Factors |  |  |  |  |  |  |  |  |
| Number of contacts | 6 [4-9] | 6 [4-9] | 6 [4-8] | 1.04 (0.92-1.17) | 0.53 | 6 [4-9] | 1.02 (0.9-1.15) | 0.76 |
| Crowding index*  <1 contacts/room  1-2 contacts/room  >2 contacts/ room | 305 (36)  397 (47)  140 (17) | 182 (35)  256 (49)  85 (16) | 29 (47)  19 (31)  14 (22) | Ref  0.51 (0.24-1.10)  0.79 (0.26-2.36) | 0.24 | 90 (36)  122 (48)  41 (16) | Ref  0.58 (0.26-1.26)  0.87 (0.27-2.87) | 0.38 |
| Contact time with index (days/ month)  ≤18 days  >18 days | 80 (10)  753 (90) | 40 (8)  478 (92) | 5 (8)  57 (92) | Ref  0.95 (0.4-2.27) | 0.91 | 35 (14)  218 (86) | Ref  1.26 (0.50- 3.18) | 0.62 |

*Definition of abbreviations*: AFB= Acid-Fast Bacilli; BMI= Body mass index

Values are median [Interquartile range] or number (percentage), unless otherwise specified

+ Univariate analysis using generalized estimating equations comparing TST converters vs. TST-positive at baseline

++ Univariate analysis using generalized estimating equations comparing TST converters vs. TST-negative

Missing data: Days per month of contact (5), hours per day of contact (5), smoking (7).

For BMI, OR estimated as the ratio of odds per 1 unit increase.
