# Supplementary material for: Incident Mycobacterium tuberculosis infection in household contacts of infectious tuberculosis patients in Brazil
Source: BMC Infect Dis. 2017 Aug 18;17:576. doi: 10.1186/s12879-017-2675-3 (PMC5563014; doi:10.1186/s12879-017-2675-3)
Supplement: Supplementary file 2 — Qualitative and quantitative analysis of tuberculin skin test (TST) and interferon gamma release assay (IGRA) results in household contacts with TST conversion according to contact age and additional TST conversion criteria. (DOCX 17 kb) [file 12879_2017_2675_MOESM2_ESM.docx]

**Supplemental Table 2:** Qualitative and quantitative analysis of tuberculin skin test (TST) and interferon gamma release assay (IGRA) results in household contacts with TST conversion according to contact age and additional TST conversion criteria.

| **Variable** | **Total** | **Household Contact Age Group (years)** | | | | |
| --- | --- | --- | --- | --- | --- | --- |
|  |  | **0-5** | **6-10** | **11-20** | **21-40** | **>40** |
| Number of contacts | 838 | 91 | 126 | 203 | 224 | 194 |
| TST <5mm at baseline | 262 | 42 | 52 | 68 | 53 | 47 |
| TST conversion Criterion 1 (Brazilian TB Program criteria) | | | | | | |
| N /total contacts (%)  TST size in converters (mm)  Median [IQR]  Mean [SD]  Range  IGRA+ in TST converters (IU/mL)  n/N (%)  Median [IQR]  Mean [SD]  Range | 56 (6.7)  17 [15-20]  17 {4.1}  [10-25]  38/52 (73)  6.41 [0.2-10]  5.6 {4.5}  [0-10] | 5 (5.5)  11 [10-20]  14 {5.3}  [10-20]  2/3 (67)  6.4 [0-10]  5.5 {5.1}  [0-10] | 11 (8.7)  19 [16-20]  18 {3.4}  [10-23]  8/11 (73)  5.9 [0.2-10]  5.6 {4.6}  [0-10] | 16 (7.8)  15 [14-18]  16 {3.3}  [10-24]  11/15 (73)  10 [0.08-10]  6.6 {4.5}  [0-10] | 12 (5.3)  18 [10-20]  17 {4.8}  [10-24]  8/11 (73)  5.8 [0.25-9.2]  4.9 {4.8}  [0-10] | 12 (6.2)  16 [15-19]  17 {4.4}  [12-25]  9/12 (75)  5.0 [0.19-10]  5.2 {4.6}  [0-10] |
| TST conversion Criterion 2 | | | | | | |
| N / total contacts (%)  TST size in converters (mm)  Median [IQR]  Mean [SD]  Range  IGRA+ in TST converters (IU/mL)  n/N (%)  Median [IQR]  Mean [SD]  Range | 54 (6.6)  15 [12-20]  16 {4.3}  [10-25]  34/50 (68)  4.7 [0.04-10]  5.0 {4.6}  [0-10] | 5 (5.5)  11 [10-20]  14 {5.3}  [10-20]  2/3 (67)  6.4 [0-10]  5.5 {5.1}  [0-10] | 12 (9.5)  18.5 [15-20]  18 {3.7}  [10-23]  8/12 (67)  5.2 [0.07-10]  5.1 {4.7}  [0-10] | 14 (6.9)  15 [14-17.5]  16 {3.3}  [10-24]  10/13 (77)  10 [0.2-10]  6.1 {4.6}  [0-10] | 13 (5.8)  16.5 [10-18.5]  16 {4.9}  [10-24]  7/12 (58)  3.6 [0.04-9]  4.5 {4.8}  [0-10] | 10 (5.2)  15 [12-16]  16 {5.1}  [11-25]  7/10 (70)  1.3 [0.01-8]  3.9 {4.5}  [0-10] |
| TST conversion Criterion 1 AND Criterion 2 | | | | | | |
| N / total contacts (%)  TST size in converters (mm)  Median [IQR]  Mean [SD]  Range  IGRA+ in TST converters (IU/mL)  n/N (%)  Median [IQR]  Mean [SD]  Range | 49 (5.8)  17 [14-20]  17 {4.2}  [10-25]  33/45 (73)  6.4 [0.2-10]  5.6 {4.5}  [0-10] | 5 (5.5)  11 [10-20]  14 {5.3}  [10-20]  2/3 (67)  6.4 [0-10]  5.5 {5.1}  [0-10] | 11 (8.7)  19 [16-20]  18 {3.4}  [10-23]  8/11 (73)  5.9 [0.6-10]  5.6 {4.6}  [0-10] | 13 (6.4)  15 [14-18]  16 {3.4}  [10-24]  9/12 (75)  10 [3.6-10]  6.5 {4.5}  [0-10] | 12 (5.3)  18 [10-19]  17 {4.8}  [10-24]  8/11 (73)  7.9 [0.04-9.3]  5.3 {4.8}  [0-10] | 8 (4.1)  15 [13-17]  17 {5.1}  [12-25]  6/8 (75)  3.6 [0.03-10]  4.7 {4.7}  [0-10] |

*Definition of abbreviations*: IGRA=. Interferon gamma release assay (Quantiferon Gold-In-Tube); IQR= Interquartile range; SD= Standard deviation

*Criterion 1* (Brazilian Guidelines): 1^st^ TST**<**10mm, 2^nd^ TST ≥10mm, and difference between 1^st^ and 2^nd^ TST ≥10mm

*Criterion 2*: 1^st^ TST <5mm, 2^nd^ TST ≥10mm, and difference between 1^st^ and 2^nd^ TST ≥6 mm

Four TST converters had missing values for IGRA: 0-5 years (2), 11-20 years (1), 21-40 years (1)

IGRA value is at time of TST conversion (8-12 weeks)
